# Adults' Awareness of Faces Follows Newborns' Looking Preferences

**DOI:** 10.1371/journal.pone.0029361

**Published:** 2011-12-21

**Authors:** Timo Stein, Marius V. Peelen, Philipp Sterzer

**Affiliations:** 1 Department of Psychiatry, Charité Campus Mitte, Berlin, Germany; 2 Berlin School of Mind and Brain, Humboldt-Universität zu Berlin, Berlin, Germany; 3 Centro interdipartimentale Mente/Cervello, Università degli Studi di Trento, Rovereto, Italy; 4 Bernstein Center for Computational Neuroscience, Humboldt-Universität zu Berlin, Berlin, Germany; University of Regensburg, Germany

## Abstract

From the first days of life, humans preferentially orient towards upright faces, likely reflecting innate subcortical mechanisms. Here, we show that binocular rivalry can reveal face detection mechanisms in adults that are surprisingly similar to inborn face detection mechanism. We used continuous flash suppression (CFS), a variant of binocular rivalry, to render stimuli invisible at the beginning of each trial and measured the time upright and inverted stimuli needed to overcome such interocular suppression. Critically, specific stimulus properties previously shown to modulate looking preferences in neonates similarly modulated adults' awareness of faces presented during CFS. First, the advantage of upright faces in overcoming CFS was strongly modulated by contrast polarity and direction of illumination. Second, schematic patterns consisting of three dark blobs were suppressed for shorter durations when the arrangement of these blobs respected the face-like configuration of the eyes and the mouth, and this effect was modulated by contrast polarity. No such effects were obtained in a binocular control experiment not involving CFS, suggesting a crucial role for face-sensitive mechanisms operating outside of conscious awareness. These findings indicate that visual awareness of faces in adults is governed by perceptual mechanisms that are sensitive to similar stimulus properties as those modulating newborns' face preferences.

## Introduction

The human face is a stimulus of outstanding social and biological relevance. From birth, humans preferentially look at faces and face-like stimuli [1]–[3]. Newborns' looking preference may reflect the evolutionary pressure to rapidly detect stimuli that exhibit shading patterns indicative of faces making eye contact with the observer [4], [5]. Since visual cortical circuits are relatively immature at birth [6], the inborn face-priority is likely mediated by a subcortical route [7]. From early infancy, such reflex-like orienting responses bias the visual system towards facial information, thereby propelling the development of highly specialized visual cortical areas associated with adults' expert skills in discriminating individual faces [8]. In turn, subcortical mechanisms may soon become inhibited by maturing cortical circuits and to finally cease to control overt behavior after the first few months of life [2]. Alternatively, these inborn biases may not be fully inhibited, but rather be counteracted by competing, newly developing cortical orienting biases [9].

Indeed, recent theories of face perception have raised the intriguing possibility that traces of the perceptual mechanisms underlying the inborn face preference remain functional in the adult visual system and continue to serve to rapidly detect faces throughout life (e.g., [7]–[8]). This is consistent with neuropsychological models of adult face perception proposing a mechanism for face detection that can operate outside conscious awareness and that most likely involves a distinct extrageniculate subcortical pathway (e.g., [10]–[12]; also see [7]). In the present study, we asked whether face detection in adults might be governed by mechanisms similar to those underlying the face-priority seen in newborns. We tested this hypothesis by examining whether perceptual mechanisms mediating visual awareness of faces in adults would be sensitive to the same properties as those influencing newborns' looking preferences.

**Figure 1:**
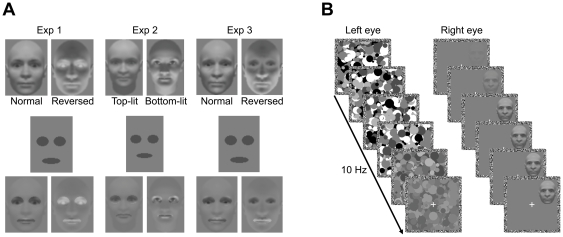
Stimuli and procedure. (A) Top row: Example test stimuli from Experiments 1–3. In Experiment 1, we compared inversion effects for faces with normal and reversed contrast polarity. In Experiment 2, we used faces that were either lit from above or from below (5). In Experiment 3, we presented faces with normal contrast polarity and “chimeric” faces with reversed contrast polarity but normal contrast polarity in the eye regions [28]. Middle row: Masks created to highlight the key features for face detection (eyes, mouth) in dark gray. Bottom row: Example stimuli with superimposed masks (at 25% transparency) illustrating the differences between test stimuli regarding the contrast relations of the key features. In (top-lit) faces with normal contrast polarity, the eyes and the mouth are dark, whereas in faces with reversed contrast polarity and in bottom-lit faces, these key features are lighter. In chimeric faces, the eyes are dark as in faces with normal contrast polarity, whereas the mouth is lighter as in faces with reversed contrast polarity. (B) Schematic of an example trial. To induce interocular suppression, high contrast CFS masks flashing at 10 Hz were presented to one eye, while a face was gradually introduced to the other eye. Participants indicated in which quadrant the test stimulus or any part of the test stimulus became visible. The contrast of the face was linearly increased over the first second of a trial, while the contrast of the CFS masks was slowly ramped down over the course of a trial.

The inborn face preference has been shown to depend on several highly specific stimulus properties. First, this face-priority cannot be accounted for by low-level stimulus properties, as newborns preferentially orient towards upright compared to inverted faces [5]. This has led to the notion of an inborn face template representing the structure of faces, such as first-order relations between features (e.g., two eyes above the mouth) that differentiate upright from inverted faces [7], [8], [13]. Moreover, newborns' looking preference for upright over inverted faces is not observed when faces are contrast-reversed or lit from below [5], thus respecting the properties that are characteristic for faces under natural viewing conditions (see Figure 1a). Importantly, newborns' upright face preference is not restricted to realistic face stimuli, but extends to simple head-shaped patterns containing only three dark blobs arranged in a head-like fashion, i.e. with the eyes above the mouth [2], [13], and this effect is not seen for contrast-reversed patterns [5].

Here, we used continuous flash suppression (CFS; [14]) to test whether the stimulus properties influencing newborns' looking preferences similarly modulate face awareness in adult observers. CFS is a variant of binocular rivalry, which has proved to be a useful tool for investigating the principles that guide the selection of conflicting input for visual awareness [15]–[19]. In CFS, high-contrast masks flashed to one eye render a stimulus presented to the other eye invisible for a couple of seconds (see Figure 1b). During such interocular suppression, activity in face-sensitive cortical areas of the ventral visual pathway is virtually abolished [20]–[23], while subcortical structures such as the amygdala continue to respond to invisible faces [21], [24], [25]. CFS therefore appears to be particularly well suited for revealing subcortical face processing in adults, as, in a sense, residual processing under CFS resembles the immature connectivity patterns in the newborns' visual system. Importantly, despite cortical face processing being strongly suppressed, upright faces overcome CFS and emerge into awareness more quickly than inverted faces [26], [27] and this inversion effect appears to be specific to faces [28]. Thus, mirroring newborns' preference for upright over inverted faces, the advantage of upright faces in gaining access to awareness may provide an ideal starting point for uncovering potential similarities in the mechanisms guiding newborns' face-priority and face detection in adults.

## Results

We tested whether the advantage of upright faces in gaining access to awareness relies on the extraction of facial properties similar to those critical for newborns' orienting biases towards upright faces. Using CFS, we rendered faces invisible at the beginning of each trial and measured the time participants needed to localize upright and inverted faces (Figure 1). If innate face detection mechanisms remain functional in adults to subserve rapid face detection throughout life (e.g., [7], [8]), we would expect similar face properties to govern awareness of faces in adults. Accordingly, we would expect the upright face advantage in overcoming CFS to be diminished for faces with reversed contrast polarity (Experiment 1) and for faces illuminated from below compared to faces illuminated from above (Experiment 2). In Experiment 3, we tested whether normal contrast polarity in the eye regions would be sufficient to elicit the upright face advantage (cf. [29]). Finally, in Experiment 4, we presented schematic face-like patterns containing three blobs that mimicked the key configuration of the eyes and the mouth, and examined the effects of inversion and contrast polarity on the duration of perceptual suppression of these stimuli during CFS.

**Figure 2:**
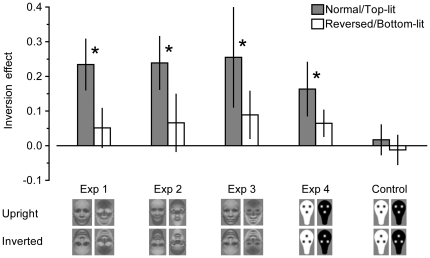
Inversion effects for all test stimulus conditions. For each subject and each condition, normalized inversion effects were obtained by dividing the difference between mean RTs for upright and inverted test stimuli by the mean RT for upright stimuli [47]. Thumbnails depict upright and inverted example stimuli for each experiment. Positive and negative error bars denote 95% confidence intervals for the comparison against zero. Asterisks indicate a significant difference between the inversion effects for normal polarity or top-lit test stimuli and reversed polarity or bottom-lit test stimuli, *p*<0.05.

Please note that absolute differences in the duration of perceptual suppression between different face conditions are difficult to interpret, as binocular rivalry is extremely sensitive to low-level differences between stimuli (e.g., [30]–[32]). As we were interested in the effect of face inversion on suppression durations *within* each face condition, we report normalized inversion effects (Figure 2) that directly quantify how much access to awareness was slowed by inversion, proportionally to each subject's suppression duration for upright faces (see Materials and Methods).

For faces with normal contrast polarity (see Figure 1a), inversion significantly prolonged the duration of perceptual suppression (Experiment 1: *t*(12)  = 6.97, *p*<0.001; Experiment 3: *t*(12)  = 3.87, *p* = 0.002; see Figure 2). This advantage of upright faces replicates previous reports [23]–[25], thus providing further evidence for the strong influence of first-order relations on face detection.

### Experiment 1: Contrast polarity

Reversing a face's contrast polarity leaves these first-order relations intact, but distorts the ordinal contrast relationships within the face (Figure 1a). As would be expected if the perceptual mechanisms underlying face detection were sensitive not only to the mere configuration of facial features but also to contrast polarity, in Experiment 1 the inversion effect for faces with reversed contrast polarity was significantly smaller than for faces with normal contrast polarity, *t*(12)  = 5.12, *p*<0.001, and failed to reach statistical significance, *t*(12)  = 1.98, *p* = 0.071 (Figure 2). This strong impact of contrast polarity may indicate that the perceptual mechanisms governing the upright face advantage are optimally tuned to detect faces under natural lighting conditions (cf. [5]).

### Experiment 2: Lighting direction

Under natural top-down lighting, the key elements of the face, i.e. the eye regions and the mouth, are darker than other facial parts. By contrast, illuminating faces from below creates a shading pattern with the eye regions and the mouth being lighter than the dark patches characteristic for faces illuminated from above (Figure 1a). Indeed, Experiment 2 revealed that the direction of illumination strongly modulated the effect of inversion on face detection under CFS. Inversion had a significantly greater influence on suppression durations for top-lit than for bottom-lit faces, *t*(12)  = 5.31, *p*<0.001 (Figure 2), and the inversion effect was significant only for faces that were lit from above, *t*(12)  = 6.82, *p*<0.001, but not for faces that were lit from below, *t*(12)  = 1.74, *p* = 0.107. The attenuation of the upright face advantage for bottom-lit and contrast-reversed faces is consistent with the notion that the mechanisms mediating privileged detection of upright faces are extremely susceptible to contrast relations within the face, possibly requiring both the eye regions and the mouth to be dark.

### Experiment 3: Contrast chimeras

Alternatively, normal contrast in the eye regions only may be sufficient to elicit an upright face advantage, as previously shown for face discrimination [29]. If so, we would expect full-blown inversion effects for contrast chimeras, i.e. faces with reversed contrast polarity but eye regions with normal contrast polarity (Figure 1a). In Experiment 3, inversion significantly prolonged suppression durations for chimeric faces, *t*(12)  = 2.84, *p* = 0.015. However, this FIE was significantly smaller than the FIE for faces with normal contrast polarity, *t*(12)  = 2.38, *p* = 0.037 (see Figure 2).

Taken together, the findings from Experiments 1–3 suggest that the perceptual mechanisms mediating the advantage of upright faces in breaking into awareness are tuned to the upright configuration of dark patches representing both the eye regions as well as the mouth. These properties match the properties that have been shown to modulate newborns' upright face preference.

### Experiment 4: Face-like patterns

In Experiment 4, we presented schematic face-like patterns containing three blobs that mimicked the key configuration of the eyes and the mouth (see Figure 2), stimuli that have previously been tested in infants [2]. Inversion of the spatial arrangement of these blobs slowed awareness for both face-like patterns with normal contrast polarity, *t*(12)  = 4.60, *p* = 0.001, as well as for face-like patterns with reversed contrast polarity, *t*(12)  = 3.73, *p* = 0.003. Crucially, however, the inversion effect was larger for face-like patterns with normal contrast polarity, *t*(12)  = 2.71, *p* = 0.019 (Figure 2). These results show that fine-grained texture and pigmentation information is not necessary for the upright face advantage. Instead, it appears that the prototypical arrangement of dark blobs representing the eyes and the mouth in upright faces is key for rapid awareness of faces during CFS, again matching newborns' looking preferences [5].

### Binocular control experiment

Finally, we tested whether faster awareness of face-like patterns with upright feature configurations are specific to CFS or whether similar effects would be observed under normal binocular viewing conditions not involving interocular suppression. For naturalistic images of faces, previous studies found the upright face advantage to be restricted to CFS and conjectured that CFS-specific unconscious processing contributed to the effect [26], [28]. Similarly, in the present binocular control experiment detection times for face-like patterns were virtually unaffected by inversion, all *t*(12) <1 (see Figure 2). Thus, shorter suppression durations for upright patterns during CFS cannot be accounted for by general differences in responding to upright and inverted face-like patterns. Rather, it appears possible that unconscious processing during CFS differentiates between invisible upright and inverted feature configurations, endowing upright face-like patterns with an advantage in entering conscious perception.

## Discussion

Previous studies have shown that upright faces initially rendered invisible by CFS gain privileged access to awareness [26]–[28]. The present results show that the perceptual mechanisms supporting visual awareness of faces do not only rely on the extraction of first-order relations between facial features, but are also highly sensitive to ordinal contrast relationships within the face. In Experiments 1–3, the upright face advantage was greatly diminished when the contrast relations characteristic for faces seen in natural environments were distorted by contrast negation or bottom-up lighting. Thus, both the spatial configuration as well as the contrast relations of the eye regions and the mouth are crucial for the privileged detection of upright faces. The attenuation of the FIE for contrast-reversed and bottom-lit faces is consistent with newborns' looking preferences that exhibit a similar sensitivity to the luminance of the eye regions and the mouth relative to the head [5].

Moreover, Experiment 4 provided direct evidence that even an abstract representation of this key information (two dark blobs above one dark blob against a lighter head shape) is prioritized for conscious awareness. Again closely resembling the looking behavior of neonates [5], simple face-like patterns consisting of dark blobs representing the eyes and the mouth against a light head-shaped background were suppressed for longer periods when the first-order relations between these blobs were inverted, and this inversion effect was attenuated for contrast negated patterns. This extends our results obtained with naturalistic face images by demonstrating that face-like feature configurations depicted in contrast relations characteristic for faces under natural top-down lighting receive priority in entering awareness even when displayed in a highly abstract manner.

These findings dovetail with a recent report showing that upright face-like patterns elicited (slightly) faster saccades than inverted patterns, but not when their contrast polarity was reversed [33]. Interestingly, no effects were obtained when observers responded manually [34]. This dissociation between saccadic and manual responses was interpreted as supporting the idea that the superior colliculus, known to trigger saccades and supposed to be the gateway to the subcortical route, mediated the bias towards face-like stimuli. Thus, in the absence of interocular suppression, rapid processing of face-like stimuli appears to be limited to the oculomotor system. This is consistent with the absence of inversion effects in the present control experiment. However, under CFS we found faster awareness of upright face-like patterns despite measuring manual responses. This suggests that reflex-like saccadic responses and release from interocular suppression may be mediated by similar mechanisms, possibly involving an extrageniculate subcortical pathway.

Indeed, under interocular suppression residual superior colliculus activity is correlated with preserved responses to invisible faces in the amygdala, another structure implicated in the subcortical face detection pathway ([21]; but see [35]). Importantly, our present study shows that the priority of face-related information, possibly embodied by this subcortical route, goes beyond mechanisms guiding reflex-like oculomotor behavior. Using CFS, a variant of binocular rivalry, we found that face-related information has a strong impact on the timing and the contents of visual awareness. Strikingly, face-related information was found to have preferential access to awareness even when presented in a coarse and highly abstract fashion.

Thus, the present study indicates a close resemblance between the facial attributes that facilitate the access of faces to awareness in adults and the facial properties that attract newborns' gaze. Our findings are in accordance with recent models of face perception suggesting that an innate face template, albeit modified by perceptual experience, continues to serve face detection throughout life [7], [8]. CFS is particularly well suited to uncover traces of an inborn face processing mechanism in adults, as it induces exceptionally strong perceptual suppression [36]. Like binocular rivalry, CFS effectively suppresses face-related activity in higher-level areas of the ventral stream, whereas activity in subcortical areas is partially preserved [21], [24], [25]. On that basis, it appears plausible to attribute unconscious readout of first-order and contrast relations to a subcortical face detection pathway [10], [11] that is supposed to assign priority to face-like visual information from birth [7].

Alternatively, recently discovered residual traces of information on invisible faces in the fusiform face area (FFA; [37], [38]), a face-selective area that has been implicated in the impact of inversion on face recognition [39], [40], might carry information about face orientation and contrast relations. However, it is currently unclear whether such residual neural responses to interocularly suppressed faces are behaviorally effective, as it has repeatedly been shown that CFS abolishes adaptation to facial attributes encoded in the ventral visual pathway [41]–[43]. While preserved ventral stream processing could still account for faster awareness of upright faces in adults, it is unlikely to account for newborns' looking preferences, as higher-level visual areas such as the FFA develop only gradually over the course of later development [44], [45]. It is important to note that even if the neural correlates of newborns' looking preferences and face detection in adults were partially distinct, it would not necessarily follow that the underlying perceptual mechanisms were different [46], [47]. In fact, the response properties of face-selective cortical circuits may initially be shaped by the inborn subcortical face detection route [7], [8], and hence could preserve the key characteristics of the innate face template despite the neural implementation being relocated.

In conclusion, using an approach pioneered by developmental psychologists, we have advanced our understanding of the facial properties that are read out in face detection and established a close link between inborn looking preferences and the perceptual mechanisms governing visual awareness of faces in adults.

## Materials and Methods

### Ethics statement

The study was approved by the Charité ethics committee and written informed consent was obtained from all participants.

### Participants

In each experiment, there were 13 participants (age range 19–42 years) with normal or corrected-to-normal vision who were naïve as to the hypotheses under investigation. Eight observers participated in both Experiments 1 and 2 and six different participants were tested in both Experiment 4 and in the binocular control experiment (in counterbalanced order, respectively). All other subjects took part in only one of the experiments.

### Apparatus and Stimuli

Observers viewed the monitor dichoptically through a custom-built mirror stereoscope, with the participants' heads stabilized by a chin-and-head rest at a viewing distance of 50 cm. Two fusion contours (9.0°×9.0° of visual angle) consisting of randomly arranged black and white pixels (width 0.5°) were displayed side by side on the screen such that one frame was shown to each eye. Test stimuli were presented against a mid-gray background within these frames. A white fixation cross (0.7°×0.7°) was drawn in the center of each frame and participants were asked to maintain stable fixation. To induce CFS, we generated high-contrast masks (8.0°×8.0°) composed of randomly arranged grayscale circles (diameter 0.4°–1.8°).

In Experiments 1–3, test stimuli were 24 faces (Experiments 1 and 3: 2.7°–3.0°×3.7°; Experiment 2: 2.1°–2.5°×3.7°; see Figure 1a), respectively, created using FaceGen Modeller 3.1 (Singular Inversions Inc., www.facegen.com), a software package widely used in face perception research (e.g., [48]). Faces were converted to grayscale and normalized to the same average luminance as the background. Inverted versions were created by flipping the images vertically. For Experiments 1 and 3, we reversed the pixel values to generate faces with reversed contrast polarity. In Experiment 2, we presented faces that were illuminated either from 66° above or from 66° below. For Experiment 3, we created “chimeric” faces by superimposing eye regions with normal contrast polarity on contrast-reversed faces [29]. The average luminance and contrast were kept constant for all face exemplars.

In Experiment 4, test stimuli were head-shaped face-like patterns (2.2°×3.7°) modeled after Farroni et al. [5]. Upright and inverted versions differed only with regard to the relative position of the three internal blobs representing the eyes and the mouth (see Figure 2). In face-like patterns with normal contrast polarity the head shape was white and the internal blobs were dark gray, whereas in face-like patterns with reversed contrast polarity the head shape was black and the internal blobs were light gray.

### Procedure

Each trial started with a 1-s fixation period. Subsequently, CFS masks changing at 10 Hz were presented to one eye and a test stimulus was gradually faded in to the other eye by increasing its contrast over the first second of each trial. Starting one second after trial onset, the contrast of the CFS masks was linearly decreased to zero over seven seconds ([27], [49]; see Figure 1b). Test stimuli were presented until response in one of the four quadrants (centered at eccentricities of 2.8°). Participants used four keys (“F”, “V”, “J”, “N”) to indicate as fast and accurately as possible in which quadrant the test stimulus or any part of the test stimulus emerged from suppression [27], [50]. Before starting the experiment, participants were shown thumbnails depicting example test stimuli from all conditions, both in upright and inverted orientations.

In Experiments 1–3, there were 192 trials (separated by a break after 96 trials), in which each combination of two test stimulus orientations (upright, inverted), two contrast conditions (normal, reversed; Experiment 2: top-lit, bottom-lit), two eyes for test stimulus presentation and 24 test stimulus exemplars was presented once. The location of the test stimulus was selected at random for each trial. Experiment 4 contained 128 trials in which all combinations of two test stimulus orientations, two contrast conditions, two eyes for test stimulus presentation and four test stimulus positions occurred equally often. Trial order was randomized.

### Binocular Control Experiment

To test whether differences between upright and inverted stimuli in overcoming suppression were specific to CFS or could be similarly found under normal binocular viewing conditions, we ran a control experiment in which we presented the same stimuli at the same positions as in Experiment 4, but displayed them binocularly. The face-like patterns were faded in transparently on top of the masks (e.g., [26], [28], [51]) and their transparency was reduced from 100% to 0% over six seconds. Participants performed the same localization task as in the CFS experiments. The control experiment consisted of 128 trials in which each combination of two test stimulus orientations, two contrast conditions and four test stimulus positions was presented equally often.

### Analysis

Only trials with correct responses (>98% in all experiments) were included in the computation of mean suppression durations. For each subject and each condition we calculated a normalized inversion effect by dividing the difference between mean suppression durations for upright and inverted test stimuli by the mean suppression duration for upright test stimuli [49]. This normalized inversion effect estimates how much localization responses were slowed by inversion and scales this inversion effect to the suppression duration for upright faces. The analysis of raw suppression durations yielded a similar pattern of results (see Text S1, Figure S1, and Figure S2).

## Supporting Information

Text S1 Results from the analysis of raw suppression durations.
(PDF)

Figure S1 Mean suppression durations from Experiments 1–3.
(TIF)

Figure S2 Mean suppression durations from Experiment 4 and mean detection times from the control experiment.
(TIF)
