# Supplementary material for: Adults' Awareness of Faces Follows Newborns' Looking Preferences
Source: PLoS One. 2011 Dec 21;6(12):e29361. doi: 10.1371/journal.pone.0029361 (PMC3244447; doi:10.1371/journal.pone.0029361)
Supplement: Text S1 — Results from the analysis of raw suppression durations. (PDF) [file pone.0029361.s001.pdf]

Adults' awareness of faces follows newborns' looking preferences

Timo Stein, Marius V. Peelen, & Philipp Sterzer

Supporting Information

In addition to the analyses of normalized inversion effects reported in the main article, we also analyzed raw mean RTs for all experiments. Again, please note that absolute RT differences between different conditions are difficult to interpret, as binocular rivalry is particularly sensitive to low-level differences between stimuli. Here we were interested in the effect of face inversion on suppression durations *within* each face condition (which were similarly estimated by the normalized inversion effects reported in the main article).

For Experiment 1, a repeated-measures ANOVA with the factors contrast polarity (normal, reversed) and orientation (upright, inverted) yielded a significant main effect of contrast polarity, $F(1, 12) = 21.90$, $p < 0.001$, reflecting shorter suppression durations for faces with normal contrast polarity, and a significant main effect of orientation, $F(1, 12) = 22.33$, $p < 0.001$, meaning that upright faces broke into awareness more quickly than inverted faces (see Figure S2A). Crucially, the interaction between contrast polarity and orientation was also significant, $F(1, 12) = 14.76$, $p = 0.002$, indicating that inversion significantly prolonged suppression for faces with normal contrast polarity, $t(12) = 6.26$, $p < 0.001$, but not for faces with reversed contrast polarity, $t(12) = 1.89$, $p = 0.083$ (Figure S2A).

For Experiment 2, a repeated-measures ANOVA with the factors illumination (top-lit, bottom-lit) and orientation revealed a similar pattern of results. For the main effect of illumination there was only a trend towards statistical significance, $F(1, 12) = 3.97$, $p = 0.069$. The main effect of orientation was significant, $F(1, 12) = 16.76$, $p = 0.001$. Most importantly, there was a significant illumination-by-orientation interaction, $F(1, 12) = 27.15$, $p < 0.001$, meaning that for top-lit faces inversion induced significantly prolonged suppression durations, $t(12) = 5.61$, $p < 0.001$, while the effect of inversion did not reach statistical significance for bottom-lit faces, $t(12) = 1.97$, $p = 0.073$ (see Figure S2B).

A repeated-measures ANOVA with the factors contrast polarity (normal, chimera) and orientation on the RT data from Experiment 3 yielded a significant main effect of orientation, $F(1, 12) = 19.22$, $p = 0.001$, and a significant interaction between contrast polarity and orientation, $F(1, 12) = 6.83$, $p = 0.023$, but no significant main effect of polarity, $F(1, 12) = 1.21$, $p = 0.292$. While inversion prolonged suppression durations for both faces with normal contrast polarity, $t(12) = 4.02$, $p = 0.002$, and for chimeric faces with reversed contrast polarity but eye regions with normal contrast polarity, $t(12) = 2.76$, $p = 0.017$, the significant interaction demonstrated that this inversion effect was larger for faces with normal contrast polarity (see Figure S2C).

For Experiment 4, a repeated-measures ANOVA with the factors contrast polarity and orientation revealed a significant main effect of polarity, $F(1, 12) = 8.29$, $p = 0.014$, with shorter suppression durations for face-like patterns with normal contrast polarity, and a significant main effect of orientation, $F(1, 12) = 14.52$, $p = 0.002$. Crucially, there was also a significant interaction between contrast polarity and orientation, $F(1, 12) = 5.37$, $p = 0.039$, meaning that although inversion prolonged suppression durations not only for face-like patterns with normal contrast polarity, $t(12) = 3.68$, $p = 0.003$, but also for face-like patterns with reversed contrast polarity, $t(12) = 2.57$, $p = 0.025$, the inversion effect was larger when the contrast polarity was normal (see Figure S3A).

Detection times in the control experiment were neither significantly modulated by contrast polarity, $F(1, 12) = 1.80$, $p = 0.205$, nor by orientation, $F < 1$, and the interaction between both factors was not significant, $F < 1$ (see Figure S3B).

In sum, the analysis of raw RTs revealed a similar pattern of results as the analysis of normalized inversion effects reported in the main article.
