# Supplementary figures and images for: Adults' Awareness of Faces Follows Newborns' Looking Preferences
Source: PLoS One. 2011 Dec 21;6(12):e29361. doi: 10.1371/journal.pone.0029361 (PMC3244447; doi:10.1371/journal.pone.0029361)

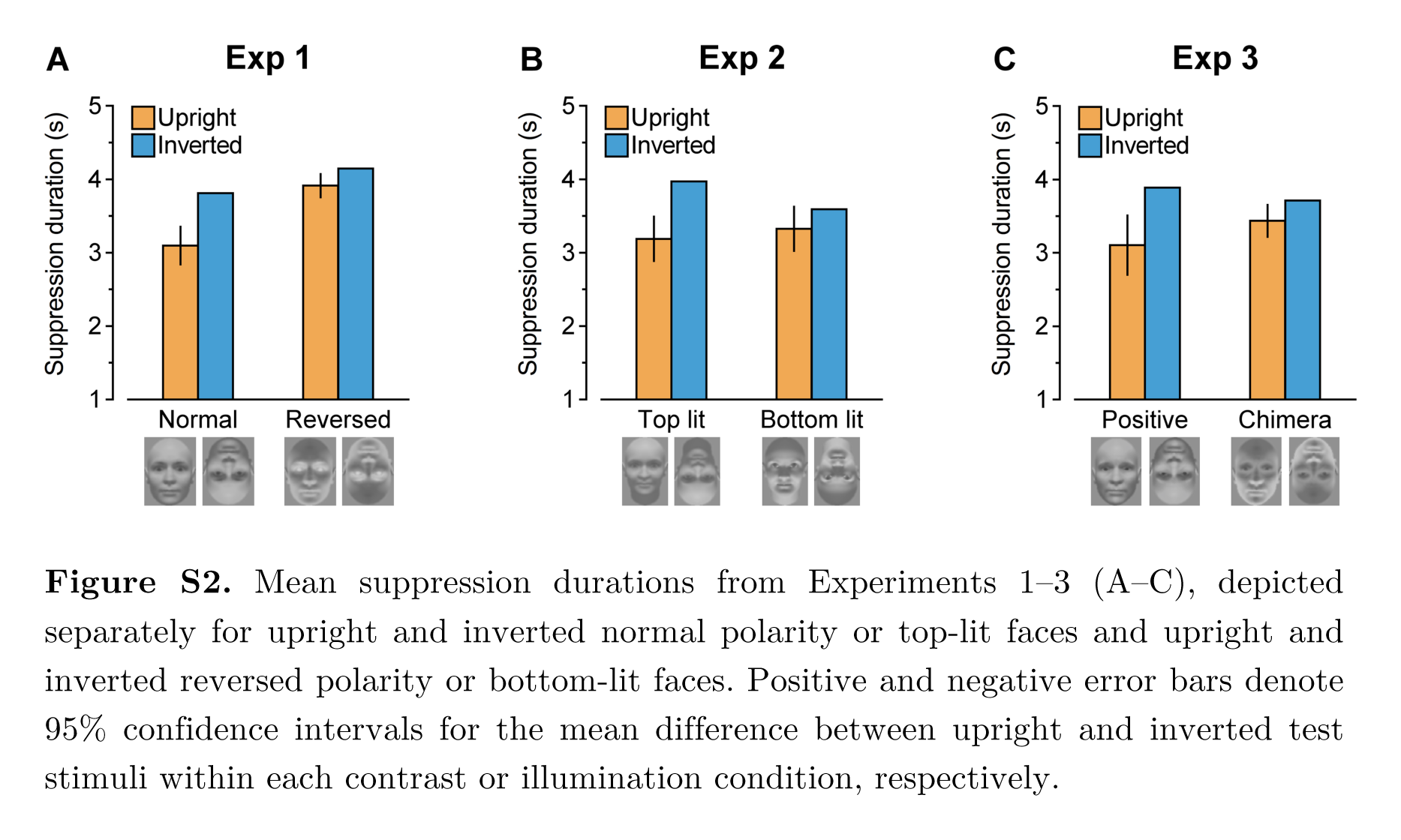

Supplement: Figure S1 — Mean suppression durations from Experiments 1–3. (TIF) [file pone.0029361.s002.tif]

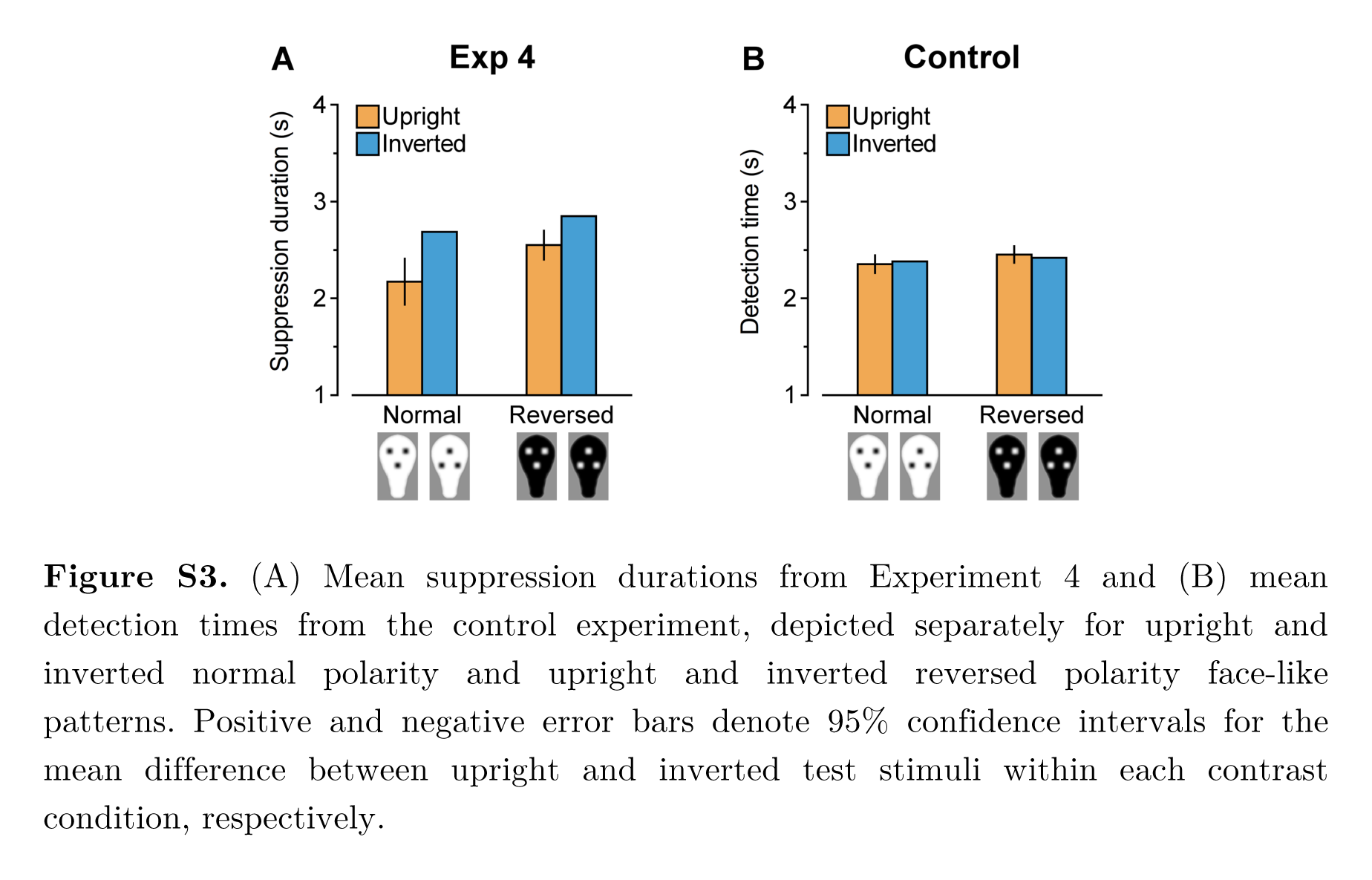

Supplement: Figure S2 — Mean suppression durations from Experiment 4 and mean detection times from the control experiment. (TIF) [file pone.0029361.s003.tif]
